# Fracture of a covered stent–graft due to heterotopic ossification of residual hematoma after endovascular treatment of superficial femoral artery pseudoaneurysm: A case report

**DOI:** 10.1097/MD.0000000000026612

**Published:** 2021-07-09

**Authors:** Jinting Ge, Tiehao Wang, Jichun Zhao, Susu Lu, Jiarong Wang, Ding Yuan

**Affiliations:** a Department of Vascular Surgery, West China Hospital, Sichuan University, Chengdu, China; b Department of Pathology, West China Hospital, Sichuan University, Chengdu, China.

**Keywords:** fracture, heterotopic ossification, pseudoaneurysm, stent–graft, superficial femoral artery

## Abstract

**Rationale:**

Endovascular treatment (EVT) is considered a preferred procedure of superficial femoral artery (SFA) pseudoaneurysm in recent years. However, heterotopic ossification (HO) after SFA pseudoaneurysm is a rare occurrence, that may cause late stent–graft fracture.

**Patient concerns:**

A 58-year-old male who underwent EVT for SFA pseudoaneurysm 8 years ago presented with a progressive mass at the right thigh and claudication. Computed tomography angiography (CTA) showed fracture and occlusion of stent–graft, which was compressed by the deep femoral artery (DFA) pseudoaneurysm and a bone-like body.

**Diagnosis:**

According to the CTA images, the stent–graft was fractured and occluded, accompanied by DFA pseudoaneurysm.

**Interventions and outcomes:**

Debridement and arterial reconstruction were performed. Pathological analysis showed that the bone-like body was derived from HO. CTA at one-year follow-up showed that the prosthetic vessel was patent and previous hematoma disappeared.

**Conclusions:**

This report demonstrates that residual hematoma can induce HO, which may result in late stent fracture, and it should thus be removed timely. Patients with SFA pseudoaneurysm who have undergone EVT should be followed up regularly.

## 1 Introduction

Development of pseudoaneurysm of lower limb arteries usually occurs due to trauma, infection, iatrogenic injury, or inflammatory process and can cause fatal or limb-threatening complications.^[1]^ Recently, covered stenting has been applied as an alternative to open surgery (OS) to exclude pseudoaneurysm of superficial femoral artery (SFA).^[2,3]^ The main advantages of endovascular treatment (EVT) are low complication rates and excellent short-term outcomes.^[4]^ Several works have reported acceptable long-term results, with 3-year patency rates ranging from 77.1% to 100%.^[1,4]^ During EVT, the hematoma cannot be cleared away, and may be absorbed spontaneously in some cases.^[5,6]^ However, the residual hematoma may lead to severe complications, such as infection and dysfunction of stent–graft.^[3]^ Here, we present a rare case of covered stent–graft fracture caused by heterotopic ossification (HO) of residual hematoma in a patient with SFA pseudoaneurysm treated by EVT 8 years ago.

## 2 Case report

**Figure 1:**
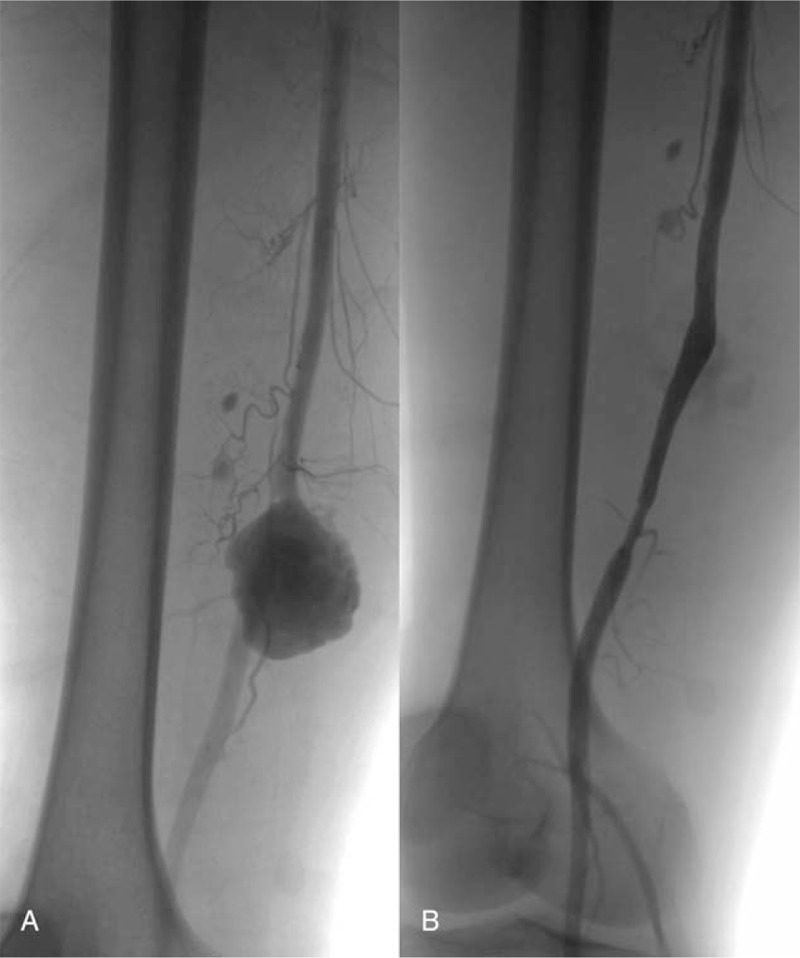
Intraoperative digital subtraction angiography 8 years ago. (A) The pseudoaneurysm was derived from the superficial femoral artery, and the adjacent bone was intact. (B) Stent–graft was patent, and no endoleaks or foreign bodies existed.

A 58-year-old man was stabbed by a sharp knife in his right thigh about 8 years ago, and he underwent debridement immediately after the stabbing in the local hospital. However, he presented with a pulsating mass soon after the debridement. He was then transferred to our hospital, and the angiogram demonstrated SFA pseudoaneurysm without defect of adjacent bone or other foreign bodies (Fig. 1A). Because this patient had already undergone debridement and no foreign bodies were shown in angiogram, an 8 mm × 70 mm self-expandable covered stent–graft (Wallgraft, Boston Scientific Corporation) was used to exclude the pseudoaneurysm. The final angiogram showed no endoleaks (Fig. 1B).

**Figure 2:**
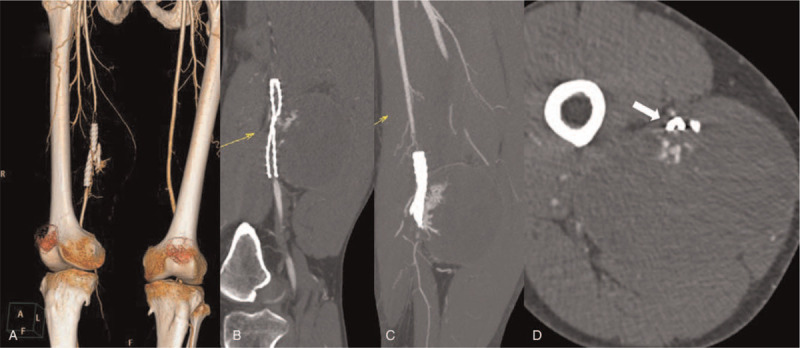
Computed tomography angiography when readmitted. (A) The stent–graft was occluded, and a material of high density was adjacent to the stent–graft. (B) The stent–graft was compressed by the pseudoaneurysm. (C) The pseudoaneurysm was derived from the side-branch of the deep femoral artery. (D) The stent–graft was fractured and occluded (white arrow).

Recently, this patient was readmitted to our institution with a complaint of a progressive mass in the right thigh and worsening symptoms of claudication. He denied drug abuse or other traumatic injuries. On physical examination, a huge tensive mass was found in the right thigh, and the skin temperature below the knee was lower than that at the contralateral limb. The pulses of ipsilateral popliteal and dorsal pedal arteries were not palpated. Computed tomography angiography (CTA) showed a 10 cm × 15 cm × 10 cm pseudoaneurysm, which was derived from a branch of deep femoral artery (DFA), and a foreign body of high density adjacent to the stent–graft. The stent–graft was fractured and occluded, and the femur was intact without any indications of fracture (Fig. 2).

**Figure 3:**
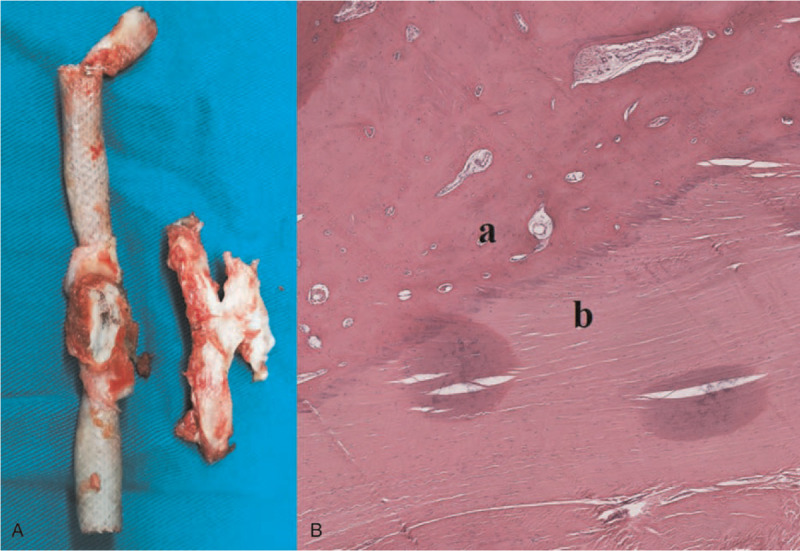
(A) Intraoperative image showing that the fabric and strut of the stent–graft were fractured, while the adjacent foreign material seemed like a piece of bone fragment. (B) Pathological analysis of the foreign material showed (a) osseous tissues without lamellar structure, which formed as a result of (b) proliferation of fibrous tissues and infiltration of inflammatory cells.

The decision to perform OS was made considering the huge pseudoaneurysm, foreign body, and fractured stent–graft. During the operation, the hematoma was cleared away, and the bleeding branch of the deep femoral artery was ligated. Visual examination of the stent–graft showed a fracture, and its fabric was torn at the fractured part. The foreign body seemed like a piece of bone fragment (Fig. 3A). The foreign body and the fractured stent–graft were extracted, and an 8 mm Intering GORE-TEX Vascular Graft was used for end-to-end bypass.

**Figure 4:**
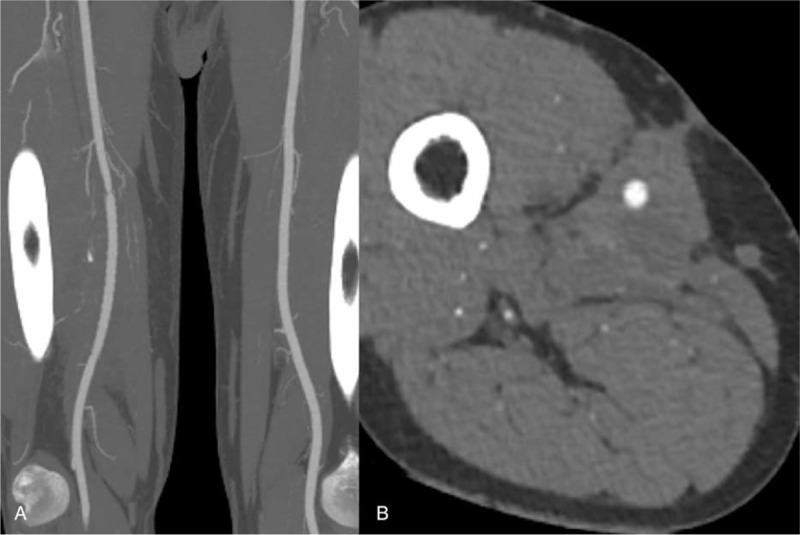
Computed tomography angiography at one-year follow-up after reintervention showes patency of prosthetic vessel and disappearance of previous hematoma.

Cultures of the explanted stent–graft and hematoma showed no signs of infection. Pathological analysis of the foreign body revealed osseous tissues without lamellar structure, accompanied by proliferation of fibrous tissues and infiltration of inflammatory cells (Fig. 3B). This result demonstrated that osseous tissues were induced from the ossification of fibrous tissues. After the operation, the patient recovered uneventfully, and the mass in right thigh and claudication quickly disappeared. He was discharged on postoperative day 5 and was treated with antiplatelet therapy to prevent restenosis. After 1 year of follow-up, CTA showed that the prosthetic vessel was patent, and the previous hematoma had disappeared (Fig. 4).

## 3 Discussion

Pseudoaneurysm of SFA resulting from penetrating trauma is relatively uncommon and is associated with substantial morbidity and mortality.^[3]^ The traditional treatment for SFA pseudoaneurysm is surgical reconstruction, which is associated with severe complications, especially for patients with multiple comorbid conditions.^[5]^ EVT has been accepted as a minimally invasive procedure for SFA pseudoaneurysm. Authors had described a high long-term primary patency of EVT for SFA pseudoaneurysm, ranging from 77.1% to 100%.^[1–4]^ Placement of a covered stent–graft has become common and is considered a treatment option for traumatic SFA pseudoaneurysm.^[1,3,7]^ Our patient was found to have developed a traumatic pseudoaneurysm 8 years ago. He had already undergone debridement, and no foreign bodies were shown in his angiogram. Therefore, EVT was a reasonable treatment option for this condition at 8 years ago. However, complications related to this procedure and their clinical effects are particularly noteworthy.^[5]^

Stent fracture is a late complication that occurs rarely and may result in artery occlusion, rupture, or pseudoaneurysm formation. Given that SFA is a dynamic anatomical segment, rigid stents are prone to compression and fracture in this area. Self-expandable stent–grafts are recommended in SFA lesion because they are flexible and can resist external deformation. However, bare-metal self-expandable stent–grafts have also been described in cases of occlusive diseases related to SFA, with a reported fracture rate ranging from 3.6% to 37%.^[8,9]^

We search literatures about EVT and SFA pseudoaneurysm since 1999 by using PubMed, and no fracture of self-expandable covered stent occurred.^[1–3,5,7,10]^ The covered stent–graft, including Wallgraft and Hemobahn/Viabahn, is composed of a self-expandable stent covered with a thin layer of polycarbonate urethane membrane and exhibits better resistance to compression and torsion than a bare-metal stent graft.^[4]^ Tielliu et al reported that the Hemobahn/Viabahn stent–graft in the region of popliteal artery has a fracture rate of 16.7%, but no fracture of self-expandable covered stent has been reported in the SFA region.^[11]^ Therefore, fracture of covered stent–grafts in SFA is rare.

Compared with conventional surgical treatment, exclusion with a covered stent–graft is a minimally invasive procedure.^[1]^ The hematoma of previous pseudoaneurysm is not cleared away during EVT, but may be absorbed spontaneously in most cases.^[1,3,5,7]^ In the present case, the high-density foreign body was demonstrated to be heterotopic bone tissues by pathological analysis. These heterotopic bone tissues, which were derived from the residual hematoma, could be products of HO. The residual hematoma is usually accompanied by organization and proliferation, and HO may occur during this process. HO is a common complication after multiple forms of extensive trauma.^[12,13]^ The branch of DFA was injured by this heterotopic bone, which caused the formation of a huge pseudoaneurysm. Furthermore, the heterotopic bone fragment was pushed close to the stent–graft by the progressive pseudoaneurysm. Eventually, the covered stent–graft was occluded and fractured because of persistent compression from this adjacent bone fragment and pseudoaneurysm. Similar cases have never been reported before, but our hypothesis was validated by imaging and pathological analysis.

Considering that this late complication was caused by residual hematoma, OS may be a reasonable choice for this condition. Our patient had a good recovery and exhibited a long-term benefit after OS. Covered stenting has been well established for SFA pseudoaneurysm, and studies have revealed that hematoma after EVT may be absorbed spontaneously.^[1,5]^ However, the residual hematoma can also cause late complications, such as infection, stent occlusion, or fracture.^[14]^ Therefore, residual hematoma needs timely removal when it persistently exists or complications occur. Moreover, this patient had no regular follow-up after EVT for SFA pseudoaneurysm, and he did not come to our hospital until the DFA pseudoaneurysm occurred and stent–graft was fractured. If he had undergone regular follow-up, the bone-like foreign body could have been discovered timely and removed before severe complications occurred. Patients with pseudoaneurysm after EVT need regular follow-up to prevent these severe complications.

The fracture and occlusion of self-expandable covered stent–graft is a rare complication after EVT. This case report demonstrates that the residual hematoma after EVT may induce HO, which can result in late stent fracture or other complications. Therefore, the residual hematoma needs timely removal when hematoma persistently exists or HO occurs. Our case also highlights the need for continuous follow-up of patients with SFA pseudoaneurysm who have undergone EVT.

## Author contributions

**Conceptualization:** Jinting Ge, Tiehao Wang, Jichun Zhao.

**Data curation:** Jiarong Wang.

**Investigation:** Jinting Ge, Jichun Zhao, Susu Lu.

**Supervision:** Jichun Zhao, Ding Yuan.

**Visualization:** Tiehao Wang, Susu Lu.

**Writing – original draft:** Tiehao Wang.

**Writing – review & editing:** Tiehao Wang, Jichun Zhao, Ding Yuan.
